# Enhancing Image Characteristics of Retinal Images of Aggressive Posterior Retinopathy of Prematurity Using a Novel Software, (RetiView)

**DOI:** 10.1155/2015/898197

**Published:** 2015-07-09

**Authors:** Chaitra Jayadev, Anand Vinekar, Poornima Mohanachandra, Samit Desai, Amit Suveer, Shwetha Mangalesh, Noel Bauer, Bhujang Shetty

**Affiliations:** ^1^Narayana Nethralaya Postgraduate Institute of Ophthalmology, Bangalore, India; ^2^i2i Telesolutions, Bangalore, India; ^3^Faculty of Ophthalmology, Maastricht University, Maastricht, Netherlands

## Abstract

*Purpose*. To report pilot data from a novel image analysis software “RetiView,” to highlight clinically relevant information in RetCam images of infants with aggressive posterior retinopathy of prematurity (APROP). *Methods*. Twenty-three imaging sessions of consecutive infants of Asian Indian origin with clinically diagnosed APROP underwent three protocols (Grey Enhanced (GE), Color Enhanced (CE), and “Vesselness Measure” (VNM)) of the software. The postprocessed images were compared to baseline data from the archived unprocessed images and clinical exam by the retinopathy of prematurity (ROP) specialist for anterior extent of the vessels, capillary nonperfusion zones (CNP), loops, hemorrhages, and flat neovascularization. *Results*. There was better visualization of tortuous loops in the GE protocol (56.5%); “bald” zones within the CNP zones (26.1%), hemorrhages (13%), and edge of the disease (34.8%) in the CE images; neovascularization on both GE and CE protocols (13% each); clinically relevant information in cases with poor pupillary dilatation (8.7%); anterior extent of vessels on the VNM protocol (13%) effecting a “reclassification” from zone 1 to zone 2 posterior. *Conclusions*. RetiView is a noninvasive and inexpensive method of customized image enhancement to detect clinically difficult characteristics in a subset of APROP images with a potential to influence treatment planning.

## 1. Introduction

Wide-field digital imaging is currently the best option in retinal imaging in infants particularly for retinopathy of prematurity (ROP) screening and documentation. However, limitations in the optics, image resolution, anatomical aspects of the infant crystalline lens, and associated features of the disease such as a rigid pupil and low contrast of the immature retina contribute to less than ideal images in some clinical scenarios. Cases of aggressive posterior ROP (APROP), an atypical form of the disease, present without the more easily discernible extraretinal fibrovascular proliferation or ridge, can be missed by the less experienced observer [1]. In these cases, vessel termination is difficult to assess, with capillary nonperfusion and ischemic beds often missed or difficult to delineate owing to the nature of the disease, the immaturity of the eye, and the resolution of the image [2]. This has led to suboptimal accuracy and agreement of diagnoses by ROP experts when based on RetCam images of the posterior pole images [3].

In the Indian scenario, APROP is a very commonly encountered entity, sometimes accounting for over 66% of all treated cases [4, 5]. Hence, there is an urgent need to address the challenge of improving the accuracy of the diagnosis of this condition. Given the limitations of image capture and acquisition, traditionally, this would require external hardware modifications of existing infant cameras or more invasive tests such as fluorescein angiography that provide clinically useful vascular information.

With advances in “image enhancing” technologies, computer-based image analysis tools have the potential to provide noninvasive, quantifiable, and objective measurements on the outputs of the available devices, in this case the RetCam (Clarity MSI, Pleasanton, USA). These technologies have been used in the interpretations of electrocardiograms and Papanicolaou smears among other clinical applications [6, 7]. Several studies have explored the diagnostic performance of these types of software by comparing their semiautomated and automated disease detection ability with a reference standard of dilated ophthalmoscopy by an experienced examiner [8–11]. Known examples include the computer-based Retinal Image multiScale Analysis (RISA), which defines quantitative parameters that reflect the curvature and diameter of vessels. It has been demonstrated to diagnose plus disease with accuracy comparable to that of human experts [12]. In addition, the Computer Aided Image Analysis of the Retina (CAIAR) allows semiautomated localization of vessels using filtered detection measurements based upon maximum likelihood estimation of vessel parameters from an image with provision for human pixel editing [13]. Wilson et al. used a total of 14 different measures for calculating tortuosity. There were satisfactory correlations of CAIAR tortuosity with expertly classified individual vessels from clinical images. While most of these types of software focus on posterior pole vascular changes and have been used to quantify disease based on vessel changes in plus disease, there have not been many studies on image enhancement or detection of disease in the peripheral retina or to highlight disease characteristics more accurately from a clinical perspective by detecting subtle features that could be either missed or less easily appreciated on clinical examination or conventional or RetCam fundus images.

We report pilot data from a novel image analysis software, which employs established mathematical algorithms but customizes it to highlight clinically relevant information. We apply this new software, “RetiView” (patent pending) to RetCam images of Asian Indian infants and discuss its utility in detecting poorly visible and undetected features in retinal images of infants with APROP.

## 2. Methods 

### 2.1. Software Protocol Acquisition

The software, “RetiView,” was developed as a research tool by i2i Telesolutions and Telemedicine Pvt. Ltd., headquartered in Bangalore, India, with technical inputs from Narayana Nethralaya Postgraduate Institute of Ophthalmology, Bangalore, India. The methods used in the software have been registered for a patent* (INDIA-3258/CHE/2011 and INTERNATIONAL-PCT/IB2012/050647)*. Noise filtering of the RetCam images was done using anisotropic diffusion filtering which allowed multilevel visualization of vascularity analysis data.

Three protocols of the software were used for clinical analysis:

1. Grey Enhanced (GE).
2. Color Enhanced (CE).
3. “Vesselness Measure” (VNM) protocol.

**Figure 1:**
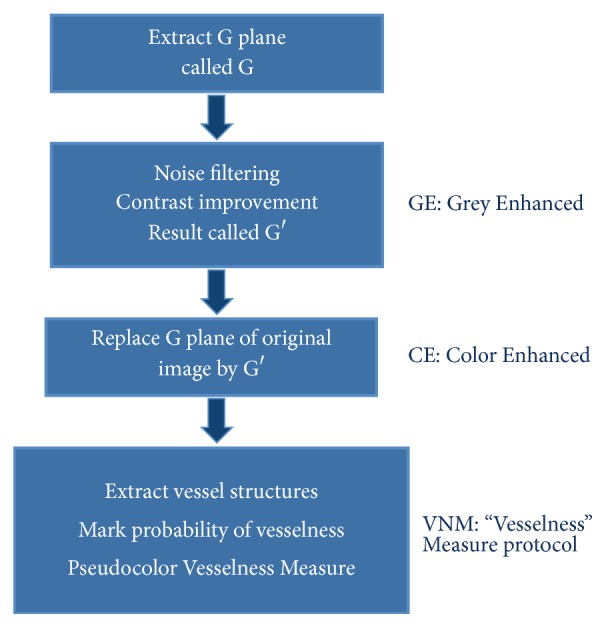
Software Protocol Acquisition.

**Figure 2:**
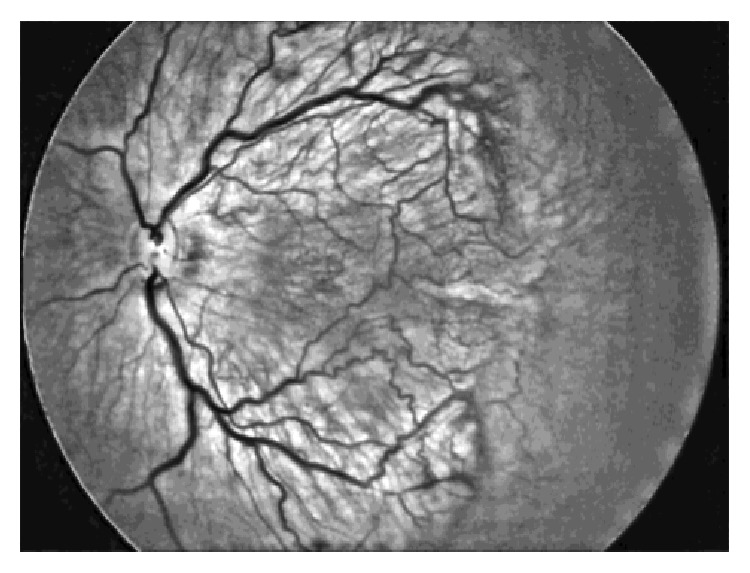
Image after contrast improvement was called “Grey Enhanced.”

**Figure 3:**
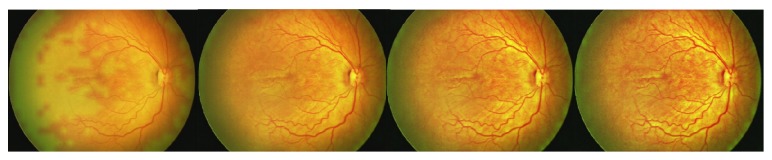
“Color Enhanced” protocol grades of 2, 4, 6, and 8.

**Figure 4:**
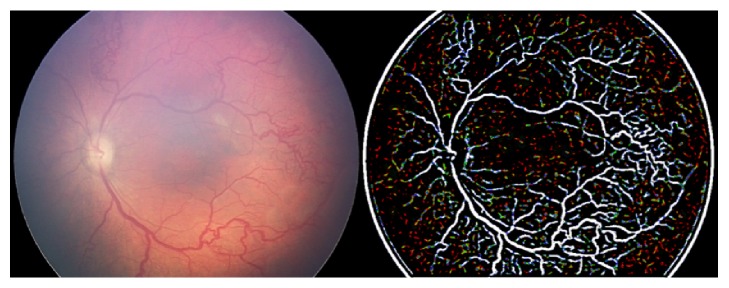
Vesselness Measure protocol applied to enhance vascular information.

Steps (summary, Figure 1) are as follows:

1. Uncompressed color images from the RetCam Shuttle (Clarity MSI, USA) were used and the Green plane was extracted (G). The G plane was chosen because it offers maximum contrast information for vessel structures.
2. Noise filtering (NF): Noise filtering was carried out on the extracted G plane. Noise filtering techniques, which preserve edge information, were used so that vessel structures could be segmented in the subsequent steps.
3. Contrast Enhancement: Contrast of the image was enhanced after filtering. Local contrast enhancement was applied in areas of 21 × 21 pixels each as vessel structures have image properties that exhibit local variations. The image after contrast improvement was called “Grey Enhanced” (GE) (Figure 2).
4. Color Enhanced protocol (CE): The image G was now replaced with G′ (i.e., enhanced Green plane, while retaining the original red and blue channels). By adjusting the channels, several grades of outputs are possible. These may detect “noise” or clinically relevant information and requires the treating physician to work in close collaboration with the software specialist to determine the appropriate grade. As this work was exploratory in nature, all grades were tried on prestudy subsets of images in a “trial-and-error” fashion to allow a relevant range of grades for testing on the study cohort. For the purpose of the study we used CE grades of 2, 4, 6, and 8 (Figure 3).
5. “Vesselness Measure” (VNM) protocol: This protocol is applied to enhance clinically useful vascular information and the output mimics a fundus fluorescein angiogram. This vessel output was mapped on a black background as linear structures using the developed algorithm. This algorithm selectively identifies “tubular structures” of different sizes and intensities (0 to 255 in 8-bit image). The vessels diameter search criteria (VDSC) were set to look for smaller vessels in images of ROP (Figure 4). The grade of VNM and VDSC was set using trial-and-error methods on a prestudy subset of images before a range for testing on the study images was decided mutually between the software specialist and the ROP expert.

**Figure 5:**
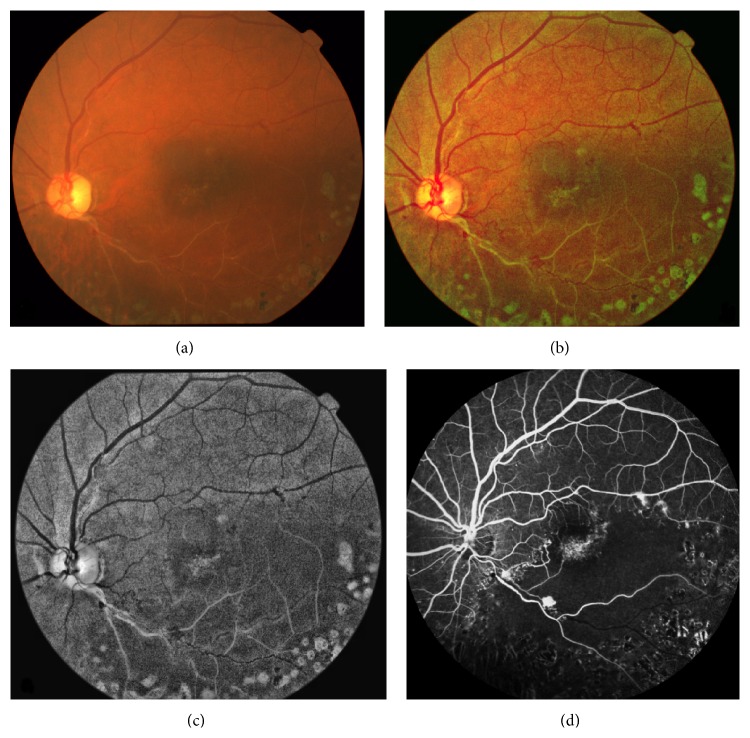
Inferotemporal branch retinal vein occlusion (a) shows that the RetiView ((b), (c)) could delineate areas of capillary nonperfusion, sclerosed blood vessels, laser scars, and neovascularization, which mimics the fundus fluorescein angiography images (d).

Prior to analyzing ROP images, we validated the algorithm by comparing processed images of common retinal conditions with fundus fluorescein angiography (FFA) images of the same. A case example of an inferotemporal branch retinal vein occlusion shows that RetiView could delineate areas of capillary nonperfusion, sclerosed blood vessels, laser scars, and neovascularization, which is similar to the FFA images (Figures 5(a)–5(d)). In particular, we were able to enhance the visibility of the neovascular (NV) fronds that may have been missed on clinical exam or routine fundus imaging, but was more visible on the GE protocol. The FFA confirmed the neovascular loops in the same location as delineated on the GE processed image. This allowed us to obtain a working range of grades used in the three protocols described above. However, in the absence of FFA validation in the ROP image set, the current output described must be regarded as a prevalidation pilot experience that converts linear tubular structures into vascular segments.

### 2.2. Clinical Protocols on APROP Images

Twenty-three RetCam imaging sessions of consecutive infants of Asian Indian origin with clinically diagnosed APROP based on the revised ICROP classification [1] were selected for the study. Clinical exam using indirect ophthalmoscopy with peripheral scleral depression was performed and served as the gold standard and was carried out by a ROP specialist. The session just prior to laser treatment was used for the study. The study met the approval of the Institute Ethics Committee and the Institutional Research Board and informed consents were obtained before every clinical procedure in all cases.

As per the routine practice at our institute, all infants underwent clinical examination and RetCam imaging prior to laser treatment. Clinical drawings were made on the file to detail the extent and characteristics of the disease and to plan the laser extent. Images of cases of APROP that required laser were also evaluated in detail and the anterior extent of the proposed laser margin, each capillary nonperfused zone, all tortuous loops, extent of abnormal or circumferential vessels, and flat neovascular fronds were documented on file [2]. The data was compared at each visit to monitor the requirement for retreatment and images were analyzed in this manner until there was complete regression of the disease. We used data from these detailed sessions as the baseline to compare the postprocessed image from the RetiView software for the following characteristics:

1. Extent of the disease (zone) determined by its anterior edge.
2. Areas discernible as “capillary nonperfusion” (CNP).
3. Tortuous loops.
4. Hemorrhages.

Images of the chosen session underwent all three protocols (GE, CE, and VNM) of the software. The same ROP specialist in a random order evaluated the postprocessed images after a period of more than six months after the laser treatment, after anonymizing patient demographic details.

The specialist graded all the APROP images for the above 4 criteria (anterior extent, CNP, loops, and hemorrhages) and the outcome was compared to the baseline data from the archived unprocessed images. The drawings of the clinical exam and “on” the pre- and postprocessed images were compared by quantifying the number and location of each of the features, namely, anterior extent of the vessels (or the margin where laser would be delivered), CNP zones (wherever visible, including within the lasered bed), loops (by marking the extent and edge), hemorrhages (location and size), and flat neovascularization (location).

## 3. Results 

Images from 23 clinically detected zone 1 APROP infants were used for this study. One eye of each baby was randomly chosen for the software analysis. All cases had comparable severity of disease in both eyes. Of these, 12 were right eye and 11 were left eye images.

Numerical data and qualitative features compared between pre- and postsoftware processed images included (1) the number of tortuous loops, (2) the location, appearance, and number of CNP areas, and (3) the number and location of hemorrhages. The location of anterior margin of the vessels was drawn over the image in each case and compared by simple superimposition of the images to determine the location of the zone of APROP (i.e., zone 1 or 2). The differential features have been summarized in Table 1.

**Figure 6:**
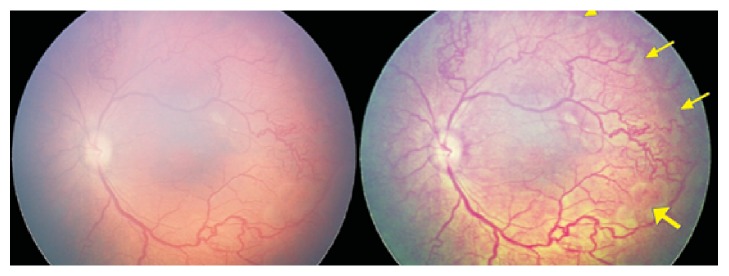
Left eye of a female infant imaged at 36 weeks postmenstrual age showing white, ischemic zones corresponding to clinically detected capillary free zones within zone 1. These irregular ischemic islands were better seen on the CE images (yellow arrows) than the RetCam image.

**Figure 7:**
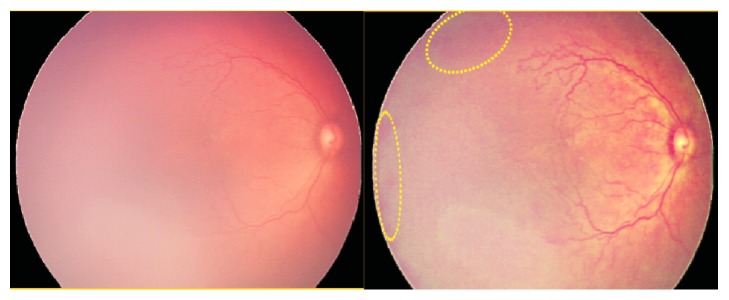
Male infant imaged at 35 weeks postmenstrual age showing clinically detected hemorrhages that could not be seen well in the RetCam image but better observed (yellow oval) in the periphery of the CE image.

**Figure 8:**
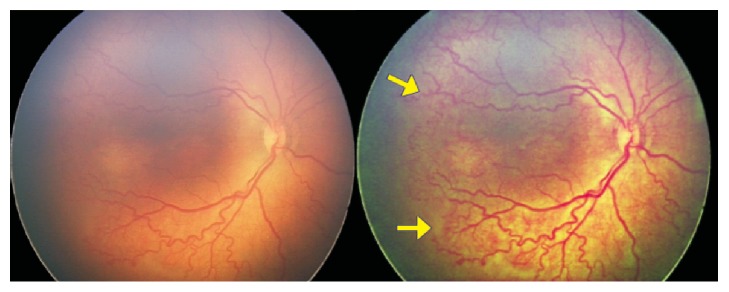
Female infant with birth weight of 1350 grams, 31-week gestational age imaged at 36 weeks postmenstrual age showing better delineation of the flat neovascular complex on the CE protocol compared to the RetCam image.

To summarize, the clinically relevant features enhanced by each of the protocols processed in the software, provided enhanced clinically relevant information by accentuating the visibility of the tortuous loops in the GE protocol by providing better contrast of the loops against the background compared to the standard RetCam image. The CE protocols provided better visualization of the “bald” zones within the capillary nonperfused zones (Figure 6). Hemorrhages that could not be seen well in the RetCam image were better observed in the periphery of the CE images (Figure 7). Neovascular fronds of the flat new vessels were also well appreciated on both GE and CE protocols (Figure 8).

**Figure 9:**
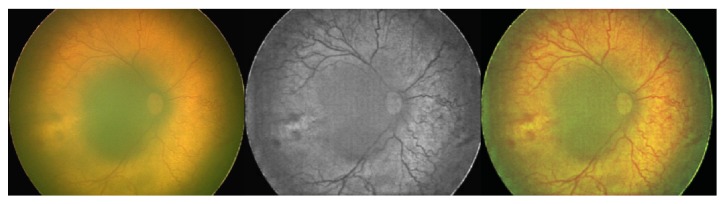
Poor pupillary dilatation in a 33-week postmenstrual age male infant obscuring the posterior pole details in the RetCam image. The GE protocol allowed better visualization of these areas and the CE protocol allowed easier observation of the nasal neovascular complex and the hemorrhages superiorly and temporally.

Despite poor pupillary dilatation and artifacts on the RetCam image, clinically relevant information was detectable on the protocol images in 2 cases (8.7%).  Figure 9 demonstrates obscured posterior pole details in the RetCam image due to poor pupillary dilatation in a 33-week postmenstrual age male infant. The GE protocol allowed better visualization of these areas and the CE protocol allowed easier observation of the nasal neovascular complex and the hemorrhages superiorly and temporally.

**Figure 10:**
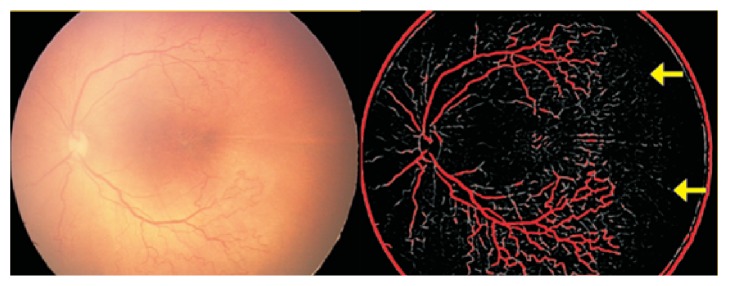
The Vesselness Measure (VNM) protocol processing shows that the anterior edge of the vessels marked as zone 1 on the RetCam image could be reclassified as zone 2 posterior based on the appearance of the smaller vessels extending more anteriorly (yellow arrows) in the processed VNM image.

The VNM protocols provided vascular information that resembled the output of an angiogram. In 3 cases (3/23, 13.04%), the anterior extent of the posterior pole vessels discerned on the VNM protocol was marked “more” anteriorly compared to the preprocessed image, causing an extension from zone 1 to zone 2 posterior (Figure 10). The software appeared to “pick up” less easily seen smaller caliber vessels lost in the poor contrast of the standard RetCam image, giving the impression of smaller capillary extensions of the primary vascular tree extending more anteriorly. This potentially allowed 13% of these images that were classified as zone 1 clinically to be potentially “reclassified” to zone 2 posterior.

## 4. Discussion

Accurate assessment and staging of the disease are essential in ensuring correct and timely treatment of ROP. Current modes are based upon clinical grading by expert examination of retinal changes, which can be influenced by the type of lens, focus, size of the optic nerve, pigmentation, and even findings in other infants examined on that day [14]. Diagnosis of ROP using RetCam imaging, albeit subjective and variable, has changed the way the disease is managed [15]. However, the image quality is not always satisfactory and is affected by the degree of magnification, motion artifacts, poor contrast, retinal pigment epithelium thickness, choroidal blood vessels, and ethnicity of the infant [16–18]. Vitreous hemorrhage, cataract, suboptimal pupil size, and hyaloid remnants may further restrict view and image quality [8]. Besides RetCam, there are other forms of digital imaging with a narrower field of view [19]. Hence, development of a quantifiable and objective diagnosis could eventually result in improved diagnostic validity and reliability. A computer-based system to allow early detection of features that suggest immediate treatment with an accuracy that is better or comparable to that of examining experts is the need of the hour.

Scientific literature has large data on the analysis and processing of fundus images, some dating back to the early 1970s [18, 20–22]. Most of these studies describe image analysis and processing to enhance details of vessel caliber and tortuosity. The steps involved are image segmentation, measurement of vessel diameter, and definition of vessel edges [23]. Image segmentation is the most important as it delineates blood vessels from the image background to allow their accurate analysis in the various algorithms. However, neonatal vascular segmentation is often difficult due to the irregularity of vessel shape and size and vessel bifurcations, crossings and choroidal vessels [18]. We used anisotropic diffusion filtering which allowed multilevel visualization of vascularity analysis in addition to noise filtering techniques, which preserve edge information, to allow segmentation of vessel structures. The VDSC were then set to look for smaller vessels in images of ROP.

In our algorithm, the GE protocol detected tortuous loops in 56.5% more easily than unprocessed images, CNP regions in 21.7%, and neovascularization and hemorrhages in 13% each. Features of the disease could also be delineated in “poor pupil” images in 8.7% of images. This is particularly important in APROP where a rigid, poorly dilating pupil is not uncommon. With the CE protocol, tortuous loops were better detected in 43.5%, capillary nonperfusion regions in 26.1%, and neovascularization and hemorrhages in 13% each. Additionally, the edge of the disease was seen well in 34.8% of images. The enhanced image on CE by providing the enhanced color contrast served to highlight these features better. The tortuous loops, for instance, revealed the “bald” or “visually empty” appearance more easily, allowing better identification. The VNM protocol allowed better “visibility” of anterior vessels in 13% of these images, which were classified as zone 1 clinically. Hence, they were potentially reclassified to zone 2 posterior, which has implications on both treatment and further follow-up.

**Figure 11:**
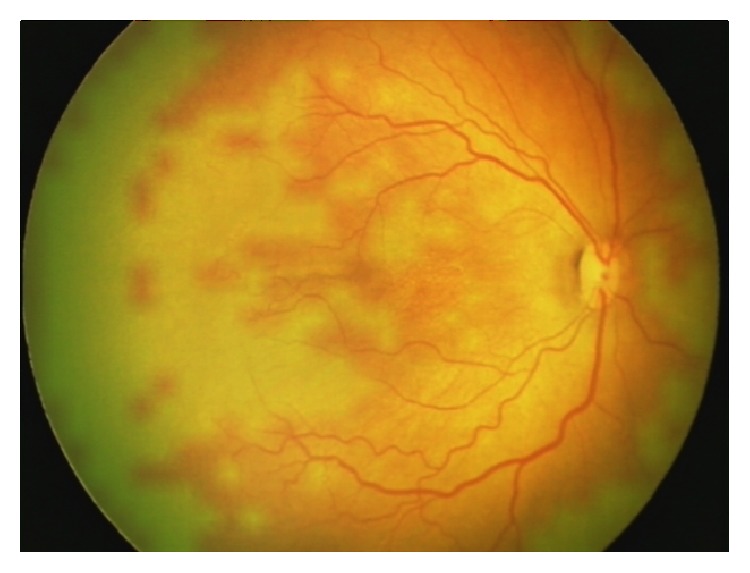
Color Enhanced protocol grade 2. The “bald” areas can be mistaken for ischemic zones. These zones are created by noise and need to be clinically discerned before interpretation is made. The appropriate grade selection is essential.

It is important to note that the principles of noise filtering and image enhancement that we have used in RetiView are well known. The unique attempt in our method is the customization of these principles to enhance clinically useful information. For example, in the CE protocols, there are 10 or more grades of outputs, which are “trial-and-error” and are an outcome of adjusting different aspects of the contrast and noise filtration (Figure 3). It would require a clinician to determine which of these shows the best clinical outcome by eliminating maximum signal noise without risking the loss of clinical detail. In grade 2, for example (Figure 11), the white spots could be wrongly interpreted as ischemic regions, when in reality they are artifacts caused by increased noise or poor filtration of the image. Hence it would require a clinician's acumen to determine which grade of which type of filtration and noise reduction would determine the optimal output. In the current version of the software, there may have been subjective bias in determining the optimal output and it is hoped that future development will allow more automated output generation. In this paper, we demonstrate the pilot work with this software. It is important to note that these outputs are therefore subject to the background pigmentation, the resolution and aspect of the image captured, the illumination and focus, and the angle of capture. Hence, before generalizability is attempted, multiuser customization must be piloted and assessed.

The current limitations of the software algorithms include the following. (1) There is an inability of the VNM to differentiate between laser marks, image edge (ora serrata), and vascular loops, since all these are picked up as “tubes.” However, the theory of continuity can be used to clinically differentiate each from the other.

(2) Raised fibrovascular proliferation is missed on VNM because of the depth of focus and the plane of imaging that is selectively enhanced by the protocols.

(3) FFA images in these cases were not available to correlate CNP. With RetCam 3, we are now acquiring FFA images of ROP to validate the software further.

The future clinical utility of this software includes accurate automated or semiautomated identification of CNP zones, choroidal vessels, edge detection of retinal boundaries and vessels, and differentiating between vascular and fibrous tissue. The VNM protocol with further refinement can identify the edge of zones, allow tracing of vessels to the ora, detect APROP zones, and make the vascular tree prominent.

In conclusion, “RetiView” is a software which demonstrates that customized image enhancement is useful in detecting clinically difficult characteristics in a subset of APROP images. By allowing better delineation of vessel characterization and zone delineation, there is a potential of influencing treatment planning. The chief advantage lies in the fact that it is a noninvasive and inexpensive method to provide vascular information, which so far could be determined on invasive tests such as angiography. Since 13% of our cases were reclassified from zone 1 to zone 2 posterior based on the VNM protocol, the software has a potential to influence the treatment plan and affect the outcome. Characterization and customization of the protocols, zone mapping, correlation with angiography, disease specific algorithms, and red and blue channel modifications need future exploration.

## Figures and Tables

**Table 1:** Summary of features that were better seen on postprocessed images compared to standard RetCam images of APROP on the 3 software protocols of RetiView (*N* = 23 eyes).

Protocol	Tortuous loops	Capillary nonperfusion regions	Neovascularization	Hemorrhage	Others
Grey Enhanced (GE)	13 (56.5%)	5 (21.7%)	3 (13%)	3 (13%)	Could see through “poor pupil” images (2, 8.7%)

Color Enhanced (CE)	10 (43.5%)	6 (26.1%)	3 (13%)	3 (13%)	Edge of the disease seen better (8, 34.8%)

Vesselness Measure (VNM)	—	—	—	—	Anteriorly visible vessels (3, 13%)
